# Antibiotic stewardship in the ICU: time to shift into overdrive

**DOI:** 10.1186/s13613-023-01134-9

**Published:** 2023-05-06

**Authors:** David Mokrani, Juliette Chommeloux, Marc Pineton de Chambrun, Guillaume Hékimian, Charles-Edouard Luyt

**Affiliations:** 1 Service de Médecine Intensive Réanimation, Institut de Cardiologie, ICAN, Groupe Hospitalier Pitié-Salpêtrière, Assistance Publique-Hôpitaux de Paris, Sorbonne-Université, Hôpital Pitié-Salpêtrière, 47-83, Boulevard de l’Hôpital, 75651 Paris Cedex 13, France; 2 Sorbonne Université, INSERM, UMRS_1166-ICAN Institute of Cardiometabolism and Nutrition, Paris, France

**Keywords:** Antimicrobial stewardship, De-escalation, Intensive care unit, Carbapenem-sparing agents

## Abstract

Antibiotic resistance is a major health problem and will be probably one of the leading causes of deaths in the coming years. One of the most effective ways to fight against resistance is to decrease antibiotic consumption. Intensive care units (ICUs) are places where antibiotics are widely prescribed, and where multidrug-resistant pathogens are frequently encountered. However, ICU physicians may have opportunities to decrease antibiotics consumption and to apply antimicrobial stewardship programs. The main measures that may be implemented include refraining from immediate prescription of antibiotics when infection is suspected (except in patients with shock, where immediate administration of antibiotics is essential); limiting empiric broad-spectrum antibiotics (including anti-MRSA antibiotics) in patients without risk factors for multidrug-resistant pathogens; switching to monotherapy instead of combination therapy and narrowing spectrum when culture and susceptibility tests results are available; limiting the use of carbapenems to extended-spectrum beta-lactamase-producing Enterobacteriaceae, and new beta-lactams to difficult-to-treat pathogen (when these news beta-lactams are the only available option); and shortening the duration of antimicrobial treatment, the use of procalcitonin being one tool to attain this goal. Antimicrobial stewardship programs should combine these measures rather than applying a single one. ICUs and ICU physicians should be at the frontline for developing antimicrobial stewardship programs.

## Background

Over the past decade, antibiotics have been prescribed in a large and steady way around the world [1–3]. Their use carries a double risk; an individual risk of adverse events [4, 5], as well as a collective risk of antibiotic resistance [6]. The former increases with each day of prescription [7], while the latter seems to be correlated with antibiotic consumption [8, 9]. Antibiotic resistance is an emerging public health threat [10]. Its consequences are dramatic, with approximately 33,000 attributable deaths per year in Europe [11], and up to 1.2 million worldwide [12]. In intensive care unit (ICU) patients, infection with resistant bacteria is a risk factor for mortality [2]. To solve or lessen the issue of antibiotic resistance, antibiotic consumption must decrease [13], especially since up to one-third of hospital prescriptions are disputable or unnecessary (viral diagnosis, treatment of a colonization, excessive duration of antibiotic therapy, etc.) [14]. Many risk factors for the emergence of resistant bacteria coexist in ICUs, making them places where antibiotic use should be as prudent as possible. In this context, antimicrobial stewardship programs (ASP) should be the forefront of efforts to control consumption in ICUs. Antimicrobial stewardship may be defined as “a coherent set of actions which promote using antimicrobials in ways that ensure sustainable access to effective therapy for all who need them” [15]. It should be viewed as a strategy to optimize antimicrobial prescribing, its main goals being to improve patient outcomes, prevent adverse events, and reduce antimicrobial resistance.

For the present review, we systematically searched the literature using the Medline database until December 1, 2022, and selected only English articles. Each opportunity developed in the present manuscript resulted from a systematic search combining the terms of interest (e.g., "antibiotic stewardship” and “antimicrobial stewardship") with the following string ("critical care" [mesh]) OR ("critical illness" [mesh]) OR ("intensive care units" [mesh]). Guidelines and literature outside the field of critical care have also been included when deemed relevant. We identified the following opportunities for reducing antibiotic consumption in the ICU: reduction of initial, empiric, antimicrobial treatment; limiting broad-spectrum empiric antibiotics; de-escalation (including the use of carbapenem-sparing agents in infections with extended-spectrum beta-lactamase (ESBL) Enterobacteriaceae strains); monotherapy instead of combination therapy for definitive treatment; dose optimization using pharmacokinetic data; and reduction of the duration of antimicrobial treatment. In this review, we will detail data on antimicrobial stewardship in ICU patients regarding these opportunities and propose recommendations for clinicians.

Although important, some topics (e.g., antifungals, antivirals, immunocompromised patients and children) were not included in our review.

## Is it possible (and safe) to reduce initial empiric antimicrobial treatment?

In 2016, the Surviving Sepsis Campaign guidelines recommended administering broad-spectrum antibiotics in patients suspected of having septic shock or sepsis, after microbiological sampling, and within 1 h [16]. Nevertheless, it is necessary to differentiate two situations that are different: septic shock, and infection (including sepsis) without shock. In case of septic shock, delaying antimicrobial treatment leads to an excess mortality rate [17, 18]. In sepsis without shock, data are less robust: Seymour et al. evaluated a 3-h sepsis bundle (i.e., blood culture, lactate measurement and antibiotic administration performed within 3 h) and found that in-hospital mortality rate increased with each hour until bundle completion. However this association was no longer found in patients without vasopressors [17]. The 2021 update of the surviving sepsis campaign clearly differentiate these two situations, introducing the possibility of withholding antibiotics if shock is absent [19]. However, these guidelines strongly advocate in favor of rapid administration of antibiotics, even if shock is absent. Nevertheless, there are data suggesting that antibiotic consumption can be even more decreased than recommended by the 2021 version of the surviving sepsis campaign. The first argument comes from published studies: in a quasi-experimental before–after study of 201 patients, the authors demonstrated that an aggressive antibiotic strategy (i.e., within 12 h of sampling and before evidence of infection) was associated with excess mortality and antibiotics use, as compared to a conservative prescribing strategy (i.e., only after evidence of infection). In this study, the median times from clinical suspicion to antibiotic prescription were 12 h and 22 h, respectively [20]. A recent before-and-after study on 1541 ICU patients, showed that an ASP (that included antibiotics withholding in patients without evidence of infection) resulted in an absolute reduction in mortality of 6.1% [21]. Last, in a randomized-controlled trial of patients with suspected community-acquired sepsis, pre-hospital antibiotic administration did not improve the prognosis of patients, as compared to antibiotic administration at hospital admission, despite a 90-min earlier antibiotic administration [22].

The second argument is that the diagnosis of bacterial infection and sepsis can be difficult, even for experienced clinicians. In a survey of 94 physicians, 88% of whom were ICU specialists with a median experience of 8 years, inter-observer agreement in identifying septic shock or sepsis was poor (Fleiss’ kappa 0.23) [23]. In the emergency department, up to one-quarter of patients admitted with suspected sepsis have proven or possible non-infectious diagnosis [24]. In another study of 2579 ICU patients, 43% of patients admitted for suspected sepsis had either no (13%) or possible (30%) infection [25]. This is also true in patients suspected of septic shock; a monocenter study found that in 25% of patients in whom septic shock was suspected, this diagnosis was refuted [26]. The last argument is that all studies that showed a relationship between time to antibiotic administration and mortality were retrospective studies on databases and included patients with sepsis and septic shock, not patients with suspected sepsis or septic shock. If there is no doubt that antibiotics are the backbone of treatment of sepsis, this is not sure for patients without sepsis: in other words, targeting only sepsis in suspected patients, clinicians could miss differential diagnosis, administer antibiotics in patients without infection and therefore expose them to undue risks.

**Fig. 1:**
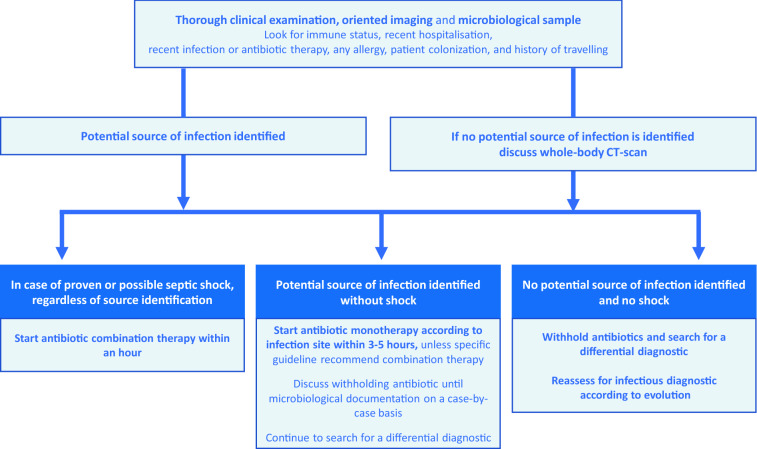
Antibiotic administration according to clinical status and source identification. *CT-scan* computed-tomography scan

Awaiting definitive proof of infection may be a reasonable attitude in patients with suspected sepsis without shock and without obvious infection. This paradigm requires that we give ourselves maximum of resources during the diagnostic process. The first step is to identify the source of infection (Fig. 1). This will guide the choice of empirical treatment and help planning surgical or interventional procedures, if necessary [27]. In an immunocompetent patient without shock, the absence of a source of infection should prompt an active search for a differential diagnosis. Microbiological samples must be systematically taken before prescribing new antibiotic, to confirm bacterial infection, obtain the pathogen responsible for infection and its antibiotic susceptibility, to adapt antimicrobial treatment. Indeed, obtaining bacteriological samples after antibiotics start may give false negative results [28]. Clinicians should not hesitate to perform invasive examinations that may help to reduce antibiotic consumption without increasing mortality [29, 30], and withhold antibiotics in colonized-but-not-infected patients, who do not benefit from antibiotics [31].

Biomarkers, and in particular serum procalcitonin (PCT), are not useful for decreasing initial antimicrobial treatment outside non-severe respiratory infection, since they do not differentiate between infectious and non-infectious inflammatory syndromes in ICU patients [32]. In the PRORATA trial, 30% of patients in the PCT-guided group had PCT value below the recommended threshold for starting antibiotics, but 70% received antibiotics, and antibiotic consumption the first day was similar in the PCT-arm and the control arm [33]. The ProACT trial showed similar result, namely an PCT-based algorithm for starting antibiotics in ICU patients did not reduce their consumption [34]. However, PCT may be useful to withhold antibiotics in patients with suspected, non-severe, community-acquired respiratory infection, such as bronchitis or acute exacerbation of chronic pulmonary obstructive disease [35].

New molecular diagnostic tools may be of interest for decreasing the antibiotic consumption in specific populations, mainly patients with suspected lower respiratory tract (LRT) infections. Razazi et al. evaluated the FilmArray Pneumonia plus Panel, a multiplex polymerase chain reaction (mPCR) assay, in patients with acute respiratory distress syndrome (ARDS) and suspected LRT infection, and found that the use of this tool helped, in patients whose mPCR was negative, to withhold new antibiotics in 60% of patients with suspected community-acquired/hospital-acquired pneumonia and in 35% of patients with suspected ventilator-associated pneumonia (VAP) [36]. More importantly, a randomized-controlled trial showed, that among patients suspected of pneumonia and in whom a broncho-alveolar lavage (BAL) was performed, the use of real-time mPCR (Unyvero Hospitalized Pneumonia Cartridge), as compared to conventional microbiological culture, led to a shorter duration of inadequate antibiotic treatment [37]. However, physicians should know that there are gaps in the panel tested, and the performance of the test does not allow us to stop or refrain from antibiotic therapy with confidence in ICU patients with suspected VAP [36, 38, 39].

In summary, whereas in patients with shock, of suspected or proven septic origin, antibiotics should not be delayed and administered as soon as possible, in some patients physicians could delay antimicrobial treatment without harm, waiting for direct examination of microbiological sample, results of imaging… The typical condition for which such a strategy may apply is VAP: in patients with suspected VAP without shock, an invasive strategy that lead to administer antibiotics only in patients with positive direct examination of broncho-alveolar lavage (BAL) fluid may decrease the rate of unnecessary antimicrobial treatment [29, 40]. The use of real-time multiplex PCR, for patients with pneumonia (community-acquired or ventilator-associated), is of interest, but the preliminary encouraging results must be confirmed before implementation of such a strategy in a meaning of antimicrobial stewardship.

## Which stewardship for empiric treatment?

### Limiting empiric broad-spectrum antibiotics

There are several opportunities to decrease empiric broad-spectrum antimicrobial treatment. The first one is to avoid broad-spectrum antibiotics in patients with community-acquired infection, when there is no risk factor for multidrug-resistant (MDR) pathogen acquisition (Table 1). Most of community-acquired infections requiring ICU admission may be treated with a non-pseudomonal beta-lactam, and even patients with early-onset hospital-acquired infection may receive non-pseudomonal beta-lactam. As an example, pathogens responsible for early-onset VAP are susceptible to non-pseudomonal 3^rd^ generation cephalosporins [41]. This class of antibiotics may be safely given in patients with early-onset VAP, in the absence of risk factors for MDR pathogens [42]. Although risk factors for MDR pathogens are mostly described for VAP/HAP (Table 1), these risk factors probably may apply for other hospital-acquired infections.

**Table 1:** Risk factors for potentially resistant pathogens responsible for ventilator-associated /healthcare-associated pneumonia

	Risk factors for MDR* pathogen	Risk factors for MRSA
North America (IDSA/ATS) guidelines [57]	Prior intravenous antibiotic use within 90 d Septic shock at time of VAP ARDS preceding VAP Current hospitalization ≥ 5 days Acute renal replacement therapy prior to VAP onset	Antibiotic treatment during the prior 90 days Treatment in a unit where the prevalence of MRSA among *S. aureus* isolates is not known or is > 20% Prior detection of MRSA by culture or non-culture screening
European and Latin American (ERS/ESICM/ESCMID/ALAT) guidelines [42]	Hospital settings with high rates (> 25%) of MDR pathogens Previous antibiotic use Recent prolonged hospital stay (> 5 days of hospitalization) Previous colonization with MDR pathogens	ICU where > 25% of the *S. aureus* respiratory isolates are MRSA

*MDR* multi-drug resistant, *MRSA* methicillin-resistant *Staphylococcus aureus*, *ARDS* acute respiratory distress syndrome, *VAP* ventilator-associated pneumonia, *IDSA* Infectious Disease Society of America, *ATS* American Thoracic Society, *ERS* European Respiratory Society, *ESICM* European Society of Intensive Care Medicine, *ESCMID* European Society of Clinical Microbiology and Infectious Diseases, *ALAT* Asociación Latinoamericana del Tórax, *ICU* intensive care unit

^*^Multidrug resistant pathogen defined as a pathogen not susceptible to at least one agent from three or more classes of antibiotics

Another issue, to limit the emergence of bacterial resistance, is to decrease empiric use of carbapenems. Indeed, even less than 3 days of carbapenem use increase the likelihood of rectal colonization with carbapenem-resistant pathogen [43]. Since carbapenems are the backbone of treatment of infections due to ESBL-producing Enterobacteriaceae, their use should be restricted to this indication. However, by definition and except in some situations, the responsible pathogen and its antibiotic susceptibility are not known when infection is suspected and antibiotics are started. Therefore, the issue is to give carbapenems in patients with a high likelihood of having infection due to ESBL pathogens. Since ESBL colonization of ICU patients range from 2.2 to 49% [44], depending on case mix and country, the empirical use of carbapenem may depend of the local epidemiology. Razazi et al. have shown that, in a cohort of ICU patients with 15% rate of ESBL colonization, only 3% of infections on ICU admission were due to ESBL-producing Enterobacteriaceae. Moreover, among the ESBL carriers, ESBL-producing Enterobacteriaceae was responsible for only 10% of first ICU-acquired infection [45]. Inclusion of ESBL colonization in the antibiotic selection process may lead to overuse of carbapenems without improvement of survival [46, 47]. The same team developed a score to identify patients who, on ICU admission, have the highest probability of being infected by ESBL-producing Enterobacteriaceae, and in whom carbapenems should be empirically given (and therefore those for whom carbapenem could be avoided) [48]. They showed that their score, based on simple variables available at ICU admission, performed better than other scores previously published [49, 50]. However, the authors did not look at the external validity of their score; therefore the variables described in their study may be different in other population with different ESBL-carriage incidence.

For ICU-acquired infection, it seems reasonable to limit carbapenem in patients with rectal or respiratory ESBL colonization, since the absence of rectal colonization in the last week has a negative predictive value of 93 to 99% for predicting infection due to ESBL-producing pathogen [51]. In patients colonized by, or at risk of *Pseudomonas aeruginosa* infection, empirical carbapenems should be avoided, piperacillin/tazobactam or an anti-pseudomonal cephalosporin being preferred.

When carbapenems are administered empirically, the use of chromogenic tests to detect ESBL may help to de-escalate sooner. Their positive and negative predictive values on urine and respiratory specimens approach 100%. If cultures retrieve Enterobacteriaceae with a negative ESBL-chromogenic test, physicians may safely discontinue carbapenems [52]. A randomized-controlled trial is underway to evaluate the benefit of a rapid antibiotic de-escalation strategy based on this test (NCT03147807 [53]). On the contrary, the performance of mPCR tests in the ICU is disappointing: the detection of the resistance mechanism of identified germs is not always reliable, and these tests do not yet allow correct guidance of empirical antibiotic therapy, particularly for *Pseudomonas aeruginosa* and ESBL-producing Enterobacteriaceae [38, 39].

Last, the use of new antibiotics (ceftazidime–avibactam, ceftolozane–tazobactam, cefiderocol) should be restricted to documented infection or suspected infection in colonized patients, when no other option exists; their use increasing the risk of emergence of resistance [54, 55].

### Avoiding unnecessary use of anti-MRSA

In recent years, rates of MRSA infections tended to decrease, even in countries with high prevalence [10, 56]. Yet, the use of anti-MRSA antibiotics remains important [1]. All recent HAP/VAP guidelines [42, 57, 58] recommended taking into account MRSA if infection occurs in unit with high MRSA prevalence (cut-offs ranging from 10 to 25%), if risk factors for MRSA carriage exist, or in case of shock for US guidelines. North American CAP guidelines and the 2021 Surviving Sepsis Campaign also recommend MRSA coverage for patients with specific risk factors (Table 1) [19, 59].

However, recent studies do not support this strategy. In 2019, Bostwick et al. evaluated the 2016 IDSA/ATS guidelines in a cohort of 3562 HAP: 99.97% of the patients included in this study should have received anti-MRSA antibiotic, whereas only 5.17% of cultures retrieved a MRSA [60]. Interestingly, in this cohort, high prevalence (e.g., > 20%) of MRSA in the care facility was not a factor associated MRSA infection, whereas it was the primary reason for the use of an anti-MRSA agent. In 2020, Jones and colleagues conducted a retrospective study including 88,605 patients hospitalized for CAP, among whom 38% received vancomycin, whereas only 4.6% of patients had positive cultures for MRSA [61]. Empirical use of an anti-MRSA agent was significantly associated with death (aRR 1.4) in the main cohort as well as in the subgroup of patients admitted to the ICU, or initially at high risk for MRSA. Use of an anti-MRSA agent was also associated with increased incidence of acute renal failure, *Clostridioides difficile* infection, and secondary Gram-negative infections. On the contrary, in HAP-setting, stopping anti-MRSA agent when cultures don’t retrieve MRSA is associated with a decrease in the rate of renal failure and length of stay without inducing an excess risk of mortality [62].

In this context it seems very important to rely on biological tests that allow not prescribing anti-MRSA agents unnecessarily, such as nasal screening for MRSA by PCR, which has a negative predictive value of 98.1% for CAP/HAP and 94.8% for VAP [63], or PCR to detect MRSA in cases of *Staphylococcus aureus* infections [64].

However, there are obviously situations where prescribing anti-MRSA agents may be not disputable, not only to target MRSA, but also other pathogens such as coagulase-negative *Staphylococci*, which may be responsible for severe device-related infections (central lines, extracorporeal membrane oxygenation cannula, etc.) or post-surgical infections in ICU patients [65].

In summary, empiric coverage of MRSA, regardless of the severity of the infection, is in the vast majority of cases unnecessary and deleterious. The actual recommendations lead to an overconsumption of anti-MRSA agents, even in high-prevalence countries [66], that may be associated with increased morbidity and mortality. The decision for empiric MRSA coverage should be done on a case-by-case basis, depending on the local ecology, known colonization, risk factors for MRSA as well as the suspected source of infection.

## Monotherapy or combination therapy?

### Combination therapy for empiric treatment

Since inadequate empiric antimicrobial treatment is associated with increased mortality in ICU patients [67], one of the main goals for physicians is to provide to patients adequate antimicrobial treatment. Adequate (or appropriate) antimicrobial treatment is defined as the use of at least one drug with an in vitro activity against the pathogen(s) responsible for infection [68].

The use of combination therapy has several theoretical advantages, including the widening of initial antimicrobial treatment spectrum. For example, most of ESBL strains remain susceptible to aminoglycosides, therefore the empirical use of an aminoglycoside in combination to a non-carbapenem beta-lactam allows to have adequate treatment in case of infection due to an ESBL-producing Enterobacteriaceae in most cases [69]. In a retrospective study including 760 patients, Micek et al. demonstrated that the addition of an aminoglycoside to a carbapenem or piperacillin–tazobactam resulted in rates of adequate empirical treatment of 94.2% and 91.4% (compared with 89.7% and 79.6% when monotherapy was used, respectively) [67]. However, combination therapy does not widen the spectrum when the pathogens are susceptible [70, 71]. Moreover, this better adequacy of empirical antibiotic treatment translates into a survival benefit only for patients with shock [72].

In summary, we recommend the use of combination therapy in empiric treatment of ICU patients with infection and shock, to increase the likelihood of coverage of pathogens responsible for infection. In these cases, except for community-acquired pneumonia, for which beta-lactam and macrolides or fluoroquinolone are recommended [59, 73], the best combination is probably a beta-lactam and an aminoglycoside.

### Combination therapy for definitive treatment

There is no clear advantage of combination therapy compared to monotherapy in the documented treatment of GNB infections, even for non-fermenting GNB (NF-GNB). In a meta-analysis published in 2014, especially when analyzing the 22 studies that compared the same beta-lactam, definitive combination therapy did not improve the prognosis of patients as compared to monotherapy [74]. However, patients included in this study had a mortality < 10%, making the translation of these results to ICU patients difficult. Other caveats of this meta-analysis were a mortality not reported in 9 studies out of 22, and the not optimal administration of aminoglycosides. Nevertheless, Adrie et al. found the same result in a cohort of 956 patients admitted to ICU for CAP, where outcomes of patients receiving beta-lactam monotherapy was similar to that of patients receiving a combination of beta-lactam and macrolide or fluoroquinolone [75]. In patients with *Pseudomonas aeruginosa* bloodstream infection or VAP, several studies found no beneficial effect of combination therapy, as compared to monotherapy, as soon as initial treatment was adequate [74, 76–78].

Although Kumar showed, in a retrospective study, that combination therapy improved survival compared to monotherapy in septic shock [79], randomized-controlled trial showed the opposite: in a trial having included patients with severe sepsis, Brunkhorst et al. showed that a combination of meropenem and moxifloxacin was not superior to meropenem alone [71].

Last, the combination of antibiotics does not prevent the emergence of resistance: a meta-analysis found that monotherapy and combination therapy led to comparable rates of colonization by resistant bacteria, but with a higher rate of superinfection in patients treated with combination therapy [74]. Another drawback of combination therapy is an increase in treatment-related adverse events [71].

However, it is possible that combination therapy may be beneficial when treating infections due to difficult-to-treat pathogens [80, 81]. Several observational studies showed a better prognosis in severe patients treated with at least two antibiotics when targeting carbapenemase-producing Enterobacteriaceae (CRE) [82–84]. However, when using the newer antibiotics, such as ceftazidime–avibactam or cefiderocol, monotherapy may be sufficient [81, 85].

In summary, we recommend combination therapy for empiric treatment of suspected or proven septic shock in ICU patients (see above) [42]. For definitive treatment, monotherapy should be used as soon as day 2–3 of antimicrobial treatment for most pathogens, when culture results and susceptibility tests are available, even for NF-GNB. Combination therapy could be discussed in patients with proven infection due to difficult-to-treat pathogens such as CRE or MDR *Stenotrophomonas maltophilia*.

## Is antibiotic de-escalation feasible and safe in ICU patients?

Antibiotic de-escalation aims to prevent the development of antibiotic resistance and to preserve carbapenems and new antibiotics. This approach consists of several measures designed to reduce antibiotic exposure and include monotherapy instead of combination therapy (see above), narrowing antimicrobial spectrum, and sparing broad-spectrum antibiotics, such as carbapenems and new beta-lactams [86].

Discontinuation of antibiotics in the absence of infection is the first step of de-escalation. Indeed, if starting antibiotics for suspected sepsis may not be disputable, particularly in case of shock, stopping antibiotics when infection is ruled out is also fundamental. This implies being able to eliminate an infection, and therefore having performed adequate bacteriological samples before introducing antibiotics (Fig. 1).

### Narrowing antimicrobial spectrum

Although it may be logical to give the antibiotic with the narrowest spectrum [87, 88], this opportunity of sparing broad-spectrum antibiotics is not systematically applied: in a multicentre observational study including 1109 patients, of whom 397 had the opportunity for de-escalation, narrowing spectrum from carbapenem to another beta-lactam or fluoroquinolone was performed in only 14.9% of then [89]. However, the safety of this measure has now been well demonstrated. Several observational studies showed no increased mortality, length of treatment and length of stay if antibiotic de-escalation is applied [90, 91]; one study even finding a better survival in patients with a de-escalation strategy, as compared to no de-escalation [90].

Leone et al. randomized 118 patients with severe sepsis to de-escalation or continuation of empirical treatment and found similar results: although patients in the de-escalation group had a longer duration of antibiotic therapy than patients without de-escalation (median antibiotic therapy of 9 vs. 7.5 days, respectively), their outcomes were similar, and patients in the de-escalation group received less anti-pseudomonal agent and less combination therapy [92]. Difference in duration in antimicrobial treatment could be explained by imbalance between groups: patients in the de-escalation group were more frequently admitted for respiratory infection and invasively ventilated. Other explanations could include psychological physician-related factors, such as physician feeling that a narrow-spectrum antibiotic should be given for a longer duration for “safety reasons”, or that at the time of de-escalation, clock should be reset at zero for calculation of treatment duration. Although these hypotheses are only speculative, it is interesting to see that another observational study found similar results; De Bus et al. found that patients with a de-escalation strategy had a longer duration of antibiotic treatment, as compared to patients with continuation of empirical treatment [93].

Unfortunately, narrowing antimicrobial spectrum does not seem to be associated with decrease of emergence of bacterial resistance, at least at short-term evaluation. In a cohort of 182 VAP patients, patients with de-escalation had trend towards decrease in the acquisition of resistant bacteria on day 21 [94]. In another cohort of 615 ICU patients, there was similar acquisition of resistant bacteria at day 14 in the de-escalation group [93]. Leone et al., in their randomized-controlled trial, found similar acquisition of MDR bacteria at day 8 [92]. Given these disappointing results, de-escalation has recently been the subject of debates [95, 96]. Gut microbiota is a human–microbial interaction system recently recognized in ICU patients [97, 98], and is the potential site of emergence of multi-resistant bacteria through antibiotic-mediated dysbiosis and weakening of colonization resistance [99]. Recent data showed that there is no linear correlation between antibiotic spectrum and dysbiosis [100]. For example, carbapenems seem to have little effect on the gut microbiota [101], whereas amoxicillin/clavulanic acid, piperacillin/tazobactam and metronidazole appear to be detrimental through their anti-anaerobe activity [102, 103]. Depending on the molecule, the duration of this detrimental anti-anaerobe effect could last several years [104]. Therefore, from an ecological point of view, the relevance of de-escalation towards molecules with anti-anaerobe potential is not resolved. Finally, it is possible that ecological impact is driven by the first days on antimicrobial treatment [105]. Thus, the benefit of starting a new antibiotic with different effects on the gut microbiota on day 3, especially if the total duration of antibiotics is short, is unclear [96]. 

### Use of carbapenem-sparing agents in ESBL infection

Data regarding the usefulness of carbapenem-sparing agents (beta-lactam–beta-lactamase inhibitor, or other non-beta-lactam agent such as fluoroquinolones) for treating patients with ESBL infection are contradictory: several observational studies [106–108], including one conducted in ICU [108], and two meta-analysis [109, 110] showed no difference in mortality rates in patients treated with carbapenem or its alternatives.

Despite these encouraging results, the MERINO trial, which included patients with Gram-negative bacteraemia resistant to third*-*generation cephalosporins, found that patients treated with piperacillin–tazobactam had an increased mortality rate, as compared to patients treated with meropenem (mortality rates were 12.3% and 3.7%, respectively) [111]. This result has been strongly criticized for several reasons. Firstly, there was imbalance in randomization with a higher rate of urinary tract infections in the meropenem group (67% versus 54.8%). Secondly, 20 of the 23 deaths in the piperacillin–tazobactam group were related to natural course of underlying disease, namely metastatic neoplastic disease or end-stage comorbidities. Last, there were some concerns with the methodology of piperacillin/tazobactam susceptibility determination: indeed, the same team showed, in a post hoc analysis, that due to technical issues with minimal inhibitory concentration (MIC) determination methods used in centers, patients were included despite a piperacillin/tazobactam MIC > 8 mg/L, and even > 16 mg/L, and that there was a correlation between mortality and piperacillin/tazobactam MIC [112]. When considering patients with strains susceptible to piperacillin/tazobactam, there was no longer mortality difference between groups. This is in line with observational studies that suggest prescribing this antibiotic when the MIC is < 8 mg/L [106, 113, 114].

Other carbapenem-sparing agents may include cefepime, fluoroquinolones, temocillin and new beta-lactam–beta-lactamase inhibitors:

- Cefepime does not seem to be a reasonable alternative for treatment of ESBL infection, since its efficacy depends on the resistance mechanism. If there is no difference between cefepime and carbapenems when Enterobacteriaceae express AmpC beta-lactamase [115], there is an excess mortality if the resistance is due to an ESBL. A randomized-controlled trial evaluating cefepime versus piperacillin–tazobactam and ertapenem in nosocomial urinary tract infections caused by ESBL-producing *Escherichia coli* was stopped early for a high failure rate in the cefepime arm [116]:
- Data on fluoroquinolones are scarce. Although there is frequent resistance [117] to this class of antibiotics in ESBL-producing Enterobacteriaceae, it seems possible to use fluoroquinolones when there is no resistance to nalidixic acid and the MIC is < 0.25 mg/L [108, 118]. Higher MICs were associated with a higher risk of death [119].
- Temocillin might be an interesting option, due to its activity on ESBL and AmpC beta-lactamase [120]; however, there are only few data available, and all are retrospective without control group [108, 121]. A randomized-controlled trial, currently recruiting, will evaluate the usefulness of temocillin in ICU patients with ESBL infection (NCT05565222 [122]).
- The new beta-lactam–beta-lactamase inhibitors (ceftazidime–avibactam, ceftolozane–tazobactam) are not used as carbapenem-sparing agents, since their ecological impact, as compared to carbapenems impact, is currently not known. Therefore, they are not recommended in this indication [81].

The use of carbapenem-sparing agents for definitive treatment of infections caused by ESBL-producing pathogens is to date restricted, in the most recent guidelines, to fluoroquinolones and trimethoprim–sulfamethoxazole for non-severe urinary tract infection [81]. For ESBL infections outside the urinary tract, carbapenem-sparing agents are not recommended [81]. Despite these recommendations, mainly due to the disputable results of the MERINO trial, some physicians still use and promote piperacillin/tazobactam as a carbapenem-sparing agent when its MIC is low, < 8 mg/L [123, 124]. We think that choosing piperacillin/tazobactam for treating ESBL infection with low MIC (< 8 mg/L) is feasible in ICU patients; but, if decided by the physician, it should be based upon multiple factors including severity of infection, control of the source and patient evolution on antimicrobial treatment.

In summary, narrowing antimicrobial spectrum is safe and feasible in ICU patients. Therefore, clinicians should promptly use the antibiotic with the narrowest spectrum once pathogen responsible for infection and its susceptibility tests are available. The use of carbapenem-sparing in ESBL-confirmed infection, although attractive, is not recommended to date, but can be let to physician’ choice for specific indications. Future studies will re-evaluate piperacillin–tazobactam and temocillin as carbapenem-sparing agents in infection outside the urinary tract. However, whether the use of these drugs, and more generally de-escalation strategy, is associated with better individual and collective ecological impact (including on gut and lung microbiota) remains to be determined [101].

## Is therapeutic drug monitoring a useful tool for antibiotic stewardship?

Pharmacokinetics of antibiotics is a key component of antibiotic therapy and antimicrobial stewardship in the ICU [125]. Indeed, due to pharmacokinetics variability in ICU patients, standardizing dosing for all patients may be problematic [126]. The DALI study confirmed this variability and the significant risk of suboptimal antibiotic dosing in the ICU [127]. In this study, 39.6% of patients treated with a beta-lactam had a plasma concentration below the MIC of pathogen responsible for infection [128–130]. Prolonged infusion and therapeutic drug monitoring (TDM) are currently recommended strategies to optimize beta-lactam therapy [19, 131, 132]. Whereas survival seems improved when continuous or prolonged beta-lactam infusion are used as compared to short-time infusion [133, 134], use of TDM during beta-lactam treatment does not seem to improve survival: indeed, a randomized-controlled trial evaluating the usefulness of TDM, as compared to no TDM, in patients receiving piperacillin/tazobactam showed similar survival in both groups [135]. A recent meta-analysis that included this trial found also no benefit from TDM-guided beta-lactam therapy [136]. Another randomized-controlled trial is currently recruiting and will help to definitively answer this question [137]. However, there are situations in which TDM may be useful: in patients treated with a beta-lactam and requiring prolonged duration of treatment [138]; when beta-lactam toxicity is suspected; in specific cases (such as ECMO patients) or when administering antibiotics such as aminoglycosides or vancomycin [125, 131], to avoid toxicity [139].

Whereas preclinical evidence and retrospective studies link suboptimal antibiotic concentrations to emergence of resistance [140, 141], no study has evaluated the impact of prolonged infusion or TDM resistance.

In summary, prolonged beta-lactam infusions after a loading dose are recommended in ICU patients. There is no argument for using TDM during beta-lactam treatment in all ICU patients. From our point of view, TDM should be reserved to severe infections requiring prolonged therapy (e.g., endocarditis), to treatment with antibiotics having narrow therapeutic window, in case of treatment failure, in specific cases (such as ECMO patients), or when antibiotic toxicity is suspected [139, 142]. The effect of pharmacokinetic optimization on the emergence of resistance remains to be determined.

## Could we reduce (even more) the duration of antibiotic treatment?

There are several arguments for moving towards shorter courses of antibiotics. The adverse effects of antibiotics increase with each day of prescription [143], including the emergence of resistant bacteria [105]. The consequences of microbiota alterations are still poorly understood, but could include an increased risk of infection in the months following treatment [144]. Two situations can be distinguished: patients with suspected but not microbiologically documented infection, and patients with documented infection.

In patients suspected of having an infection, but in whom quantitative cultures, when sampling was performed before antibiotics are started, are negative or at a non-significant concentration, antibiotics should be discontinued. In a cohort of 89 patients with suspected VAP but with negative quantitative cultures of BAL fluid, Raman et al. evaluated an early discontinuation antibiotic strategy [31]. They showed similar mortality in the early discontinuation group, despite shorter duration of antibiotic therapy (4 days vs. 9 days). The rate of emergence of multidrug-resistant bacteria was significantly lower in this group (7.5% versus 35.7%).

When infection is microbiologically documented, treatment duration depend on the site of infection and the pathogen. Table 2 summarizes the main studies that demonstrated the non-inferiority of short- vs. long duration of antimicrobial treatment.

**Table 2:** Studies showing that short-course antibiotic regimens are non-inferior to long-course antibiotic regimens

Infection site	Short-course regimen (days)	Long-course regimen (days)	References
Uncomplicated Gram-negative bloodstream infection	7	14	[157]
Acute exacerbation of COPD	= < 5	> = 7	[187]
Community-acquired pneumonia	3–5	7–10	[188–191]
HAP and VAP	7–8	14–15	[146, 147]
Intra-abdominal infections and post-operative peritonitis	4–8	10–15	[192, 193]
Severe community-acquired UTI	5–7	10–14	[194–198]
Cellulitis	5–6	10	[199–201]
Neutropenia	3 days of apyrexia and clinical recovery	3 days of apyrexia and clinical recovery and neutrophil count > 0,5 × 10^9^ cells/L	[202]
Osteomyelitis	42	84	[203]

*COPD* chronic obstructive pulmonary disease, *HAP* healthcare-associated pneumonia, *UTI* urinary tract infection, *VAP* ventilator-associated pneumonia

For VAP, all guidelines recommended an 8-day course of antimicrobial treatment, including when the causative pathogen was a non-fermenting Gram-negative bacilli [42, 57, 145]. These guidelines were mainly based on the results of the PneumA trial, that demonstrated the non-inferiority of an 8-day versus 15-day course of antibiotics in the treatment of VAP [146]. In that study, no difference in mortality was found between the two groups, and the recurrence rate was identical except for *Pseudomonas aeruginosa* (32.8% vs. 19% in the 15-day group). Interestingly, recurrences or superinfections were less often caused by multidrug-resistant germs in the 8-day group. This result has since been confirmed by another randomized-controlled trial [147] and a meta-analysis [148]. However, recently Bouglé et al, evaluated the possibility of an 8-day versus 15-day antibiotic treatment for *Pseudomonas aeruginosa* VAP. This randomized, open-label, multicentre trial was stopped after 2 years because of lack of recruitment, was therefore underpowered, and failed to demonstrate the non-inferiority of the 8-day treatment on a composite endpoint that combined mortality and recurrence of lung infection [149]. Following these results, some advocated for a 14-day course of antimicrobial treatment for NF-GNB VAP [150], whereas others advocated for an 8-day course of antibiotics, arguing that besides differential time at risk bias in the iDiapason and PneumA trials [146, 149], patients with short duration of treatment have similar outcomes and less antibiotics exposure [151]. From our point of view, duration of treatment of VAP should be set at 7 days, whatever the pathogen responsible for infections, since harms of long course of antibiotics probably overweigh its disputable benefits on relapse. This recommendation may perhaps not apply in patients with severe SARS-CoV-2 pneumonia and recurrent VAP episodes: indeed, it has been shown that COVID-19 patients had increased VAP rates, with multiple episodes, even occurring during antimicrobial treatment of the previous VAP episode [152, 153]. In these patients with multiple VAP recurrences, the choice of non-conventional or not recommended therapies (prolonged duration of treatment, combination therapy using IV or nebulized antibiotics) or procedures (systematic bacteriological sampling at the end of theoretical end of treatment) should be discussed on a case-by-case basis, the best strategy remaining to be determined.

Although retrospective studies having evaluated the duration of treatment for Gram-negative bacteraemia were conflicting [154–156], a randomized-controlled trial in patients with Gram-negative bacteraemia, who were stable and apyretic after 48 h, showed the non-inferiority of a 8-day over a 14-day course of antimicrobial treatment on a primary composite endpoint that included all-cause mortality, relapse, local suppurative or distant complications and readmission or extended hospital stay [157]. The results of another similar trial, currently recruiting, will confirm or not these results (BALANCE trial, NCT03005145 [158]). Short duration of treatment for bacteraemia is not always possible, since actual recommendations emphasize the need for a 14-day course of antimicrobial for uncomplicated *Staphylococcus aureus* bacteraemia. However, several observational studies suggested a shorter duration of treatment may be harmless in the absence of endocarditis, sustained bacteraemia or persistent fever, metastatic infection, or implanted prosthesis [159, 160]. A randomized-controlled trial is currently recruiting to test the non-inferiority of a 7-day course for uncomplicated *S. aureus* bacteraemia, as compared to a 14-day course (NCT03514446 [161]). Awaiting these results, the choice of shortening duration of treatment of uncomplicated *S. aureus* bacteraemia to 7 days may be discussed case be case, but seem possible and sometimes pertinent, even in ICU patients.

### Usefulness of biomarkers to shorten the duration of antimicrobial treatment

The use of PCT in the ICU is another strategy that can reduce the duration of antibiotic therapy: indeed, it could be logical to guide duration of antimicrobial treatment on the intensity of systemic inflammatory response: if the duration of the inflammatory response is absent or short, it might be logical to shorten duration of antibiotics. Since the PCT blood level is related to the intensity of systemic inflammatory response to infection, it might be logical to adapt the duration of antibiotics on PCT kinetics during treatment. Bouadma et al. showed that the use of a PCT-based algorithm to stop antibiotics allowed reducing antibiotic exposure without increasing mortality. Patients in the PCT group had more antibiotic-free days than those managed in the conventional group (14.3 versus 11.6 days) [33]. The results of this study have been confirmed in a meta-analysis including 6,708 patients suffering of respiratory tract infection, of whom 2,447 were in the ICU. In this meta-analysis, the use of a PCT-based algorithm allowed to reduce the duration of antibiotic (8.1 days versus 9.5 days) without harm, and with fewer antibiotic-related adverse events [162]. Therefore, a PCT-based algorithm could be used as one tool to shorten duration of antibiotics in the ICU. Whether or not such a strategy has to be implemented in an antimicrobial stewardship program depends on each physicians’ believes and willingness, and to the strategy already applied: if short (< 7 days) durations of antibiotics are systematically applied, the use of a PCT-based algorithm should no decrease dramatically antibiotics consumption.

Regardless of the strategy chosen, consideration of the duration of antibiotic treatment should be included in a clinical approach, where source control remains paramount [27]. In summary, for most infections in the ICU, duration of antimicrobial treatment could be set at 7 days, and may be even shortened using biomarkers such as PCT. Table 3 summarizes our proposed duration for most situations in ICU patients.

**Table 3:** Proposed duration of antimicrobial therapy according to infection site

Infection site	Proposed duration (days)	References
Uncomplicated bloodstream infection	7–8	[204]
Uncomplicated catheter-related infection	7, unless *Staphylococcus aureus* and *Candida* spp.	[205]
Acute exacerbation of COPD	5	[206]
Community-acquired pneumonia	5–7	[59]
HAP and VAP	7–8, including *Pseudomonas aeruginosa*	[42, 57, 145]
Intra-abdominal infections	5–7, with optimal source control	[192]
Post-operative peritonitis	5–15, with optimal source control	[192, 193]
Severe community-acquired UTI		[207]
Female	7	
Male	14	
Cellulitis	7	[208]
Necrotizing fasciitis	14–21, with full surgical debridement	[208]
Neutropenia	3, if apyrexia and no documentation in a stable patient	[209]

*COPD* chronic obstructive pulmonary disease, *HAP* healthcare-associated pneumonia, *UTI* urinary tract infection, *VAP* ventilator-associated pneumonia

## What is the impact of antibiotic stewardship programs?

The principles presented in this review are intended to help achieve stewardship goals (improve outcome, prevent adverse events, reduce antimicrobial resistance) in the ICU. They are associated with better outcomes [163], and may be integrated, totally or partly, into an ASP (Table 4).

**Table 4:** Bedside principles for optimizing antibiotic prescribing in the ICU

Carry out a thorough clinical examination with oriented imaging ± whole-body CT scan Use invasive diagnostic tools, especially if the patient is severe on admission. Microbiological sampling is mandatory prior administering antibiotic
If septic shock is suspected, use broad-spectrum combination therapy within one hour
Without shock, if a potential source of infection is identified, use monotherapy unless specific recommendation (e.g., community-acquired pneumonia)
Without shock, if sepsis is suspected and no source of infection identified, withhold antimicrobial treatment. Search for differential diagnosis
Empiric antibiotic therapy should be selected based on identified source and local ecology Limit the use of carbapenems to patients with a high likelihood of ESBL infection. Use of rectal or respiratory ESBL colonization may be useful
Systematically reassess antibiotic therapy after 48 h
De-escalation should be done as early as possible. For early de-escalation, ESBL-chromogenic tests may be useful
In the absence of documentation after 48 h, search for a differential diagnosis
In most cases, the definitive treatment should be a monotherapy. Combination therapy can be discussed for difficult-to-treat pathogens or specific localizations (endocarditis, prosthetic device infection, joint and bone infection, abscess)
Use prolonged beta-lactam infusion after initial loading dose in severe patients (e.g., shock)
TDM is recommended for aminoglycosides and vancomycin, and in general for antibiotics having narrow therapeutic window or suspected drug toxicity Beta-lactams TDM should be used for prolonged therapy and in specific situations (augmented renal clearance, renal replacement therapy, ECMO)
Use short-course (7-day) for most of infections. PCT may be useful to help shorten the duration of antimicrobial treatment

*CT* computed-tomography, *ECMO* extracorporeal membrane oxygenation, *ESBL* extended-spectrum-beta-lactamase, *ICU* intensive care unit, *PCT* procalcitonin, *TDM* therapeutic drug monitoring

Implementing ASP makes it possible to reduce antibiotic use without increasing mortality. [164, 165]. Moreover, it significantly reduces the incidence of infections and colonization with MDR bacteria and *Clostridioides difficile* infections [21, 166, 167].

However, in a recent survey of 113 French ICUs, only 54% of respondents stated that they followed local antibiotic protocols, and 43% were familiar with the term antimicrobial stewardship [168]. Therefore, it is critical to focus on the means to implement ASP.

ASPs can combine three types of interventions: restrictive (formulary restrictions, specialist preauthorization), incentive (prospective audit and feedback, education), and organizational (multidisciplinary team approach, antimicrobial stewardship meeting) [169, 170].

Several observational studies have shown that restricting prescriptions is an effective measure to reduce broad-spectrum antibiotics use, and is associated with a reduction in antimicrobial resistance [171]. A recent meta-analysis found a significant effect of restrictive fluoroquinolone or piperacillin/tazobactam prescribing on short-term resistance emergence, particularly in high-resistance settings [172]. This could be particularly useful when using new antibiotics with emerging resistance [173–175]. In a before-and-after study, Le Terrier et al. observed a decrease in the rate of acquisition of ESBL-producing Enterobacteriaceae after the implementation of a restrictive antibiotic prescription strategy, including the start of antibiotics only when sepsis was microbiologically documented [21]. The proportion of patients not receiving antibiotics was higher after the implementation (53.2% versus 42.1%), which may have contributed to decrease in ESBL acquisition.

Feedback and prospective audit are strategies that have also proven to reduce antibiotic use and prevent resistance in the ward or in the ICU [176–178]. A recent meta-analysis confirmed the safety of these measures [164].

Finally, multidisciplinary rounds (infectious disease specialist, microbiologist, pharmacist) in ICUs are also effective [179–181]. Their effect depends on patient complexity (e.g., greater effect in complex patients). Interestingly, such interventions may have lasting effects through learning [179]. Frequency and modalities of these interventions should be discussed according to local resources.

Improvements in stewardship are urgently needed, and the measures discussed here should be more widely implemented [182].

## Perspective for future research

The aim of this review was to describe different interventions that can be used in clinical practice to optimize antibiotic use in the ICU. However, several points developed in the present review are clearly under-investigated and merit further studies.

Firstly, rapid diagnostic tools (PCR, chromogenic tests, etc.) for detection of pathogens and their resistance are a promising way to reduce antibiotic consumption [37]. Randomized controlled trials are warranted to confirm this, assess their safety and efficacy in reducing antibiotic consumption, alone or in a more general ASP.

Secondly, the real ecological impact of antibiotic de-escalation has been poorly investigated. As an example, the true impact of de-escalating from a carbapenem to piperacillin/tazobactam on the microbiota and on the outcome is not known. [101]. Results obtained in healthy subjects are not directly applicable to critically ill patients due to differences in baseline microbiota, and should therefore be studied in ICU patients [97, 103] [100, 183, 184]. Furthermore, the reality of the impact of short courses of broad-spectrum antibiotics should be compared to that of de-escalation. Whether or not short or ultra-short antibiotic courses without de-escalation could be of interest to limit the emergence of resistance at the individual and ICU level remain to be determined [95, 96]. Future studies should focus on long-term impact of de-escalation.

Thirdly, TDM in the ICU is another topic of interest. Based on recent studies, TDM cannot be universally recommended because it does not improve patient outcomes. However, it is possible that improvements in techniques, if they allow rapid results and real-time use, could beneficiate to patients.

Finally, although not discussed in this literature review, non-antibiotic anti-infective methods may become a serious alternative in the coming years [185, 186].

## Conclusion

**Fig. 2:**
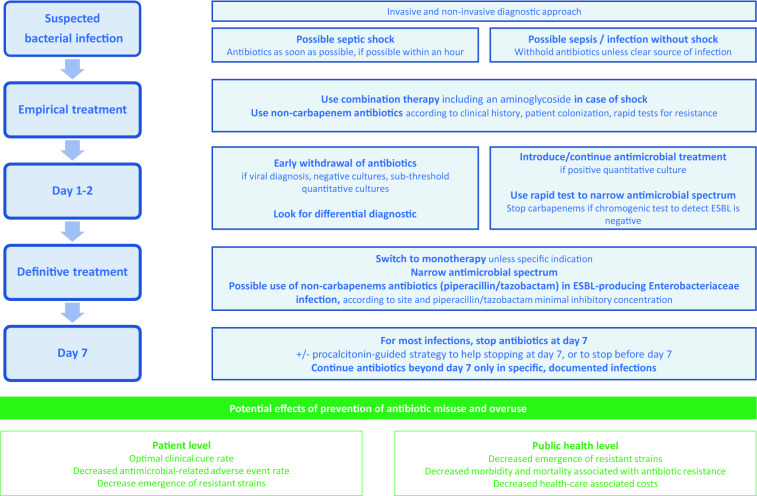
Proposed algorithm to decrease antimicrobial consumption in the ICU (in blue) and potential beneficial effects of reducing antibiotics consumption (in green). *ESBL* extended-spectrum beta-lactamase

Given the huge antibiotic consumption in the ICU, and the easiness for ICU physicians to document infection before giving antibiotics, ICUs should be at the forefront for ASPs, and ICU physicians should be leaders in their hospital for such programs. There are several opportunities to decrease antibiotic consumption in the ICU; the measures that could be easily implemented include the following (Fig. 2 and Table 4): refraining from immediate prescription of antibiotics when infection is suspected (except in patients with shock, where immediate administration of antibiotics is essential); limiting empiric broad-spectrum antibiotics (including anti-MRSA antibiotics) in patients without risk factors for MDR pathogens; switching to monotherapy instead of combination therapy and narrowing antimicrobial spectrum when culture and susceptibility tests results are available; limiting the use of carbapenems and new beta-lactams; and shortening the duration of antimicrobial treatment, the use of procalcitonin being one tool to attain this goal. ASPs should combine these measures rather than applying a single one. However, implementing an ASP mostly depends on physician willingness to decrease its antimicrobial consumption. Moreover, ASPs should integrate measures regarding antifungal stewardship.

Future research should be perform on new tools (rapid tests for pathogens or resistance, molecular tests, etc.) that may allow a quicker identification of pathogens responsible for infection and their resistance to antimicrobials. If clinically relevant, namely allowing clinicians to de-escalate sooner and spare broad-spectrum antibiotics, these tools should be implemented in future antimicrobial stewardship programs.

## Data Availability

Not applicable.

## Abbreviations

ARDS: Acute respiratory distress syndrome
ASP: Antimicrobial stewardship program
BAL: Bronchoalveolar lavage
CAP: Community-acquired pneumonia
CRE: Carbapenemase-producing Enterobacteriaceae
ESBL: Extended-spectrum beta-lactamase
HAP: Hospital-acquired pneumonia
ICU: Intensive care unit
LRT: Lower respiratory tract
MDR: Multidrug resistant
MIC: Minimal inhibitory concentration
MRSA: Methicillin-resistant *Staphylococcus aureus*
NF-GNB: Non-fermenting Gram-negative bacilli
MV: Mechanical ventilation
mPCR: Multiplex polymerase chain reaction
PCT: Procalcitonin
SAPS: Simplified Acute Physiology Score
SOFA: Sequential Organ Failure Assessment
VAP: Ventilator-associated pneumonia
